# Modulation of Cytokines and Immune Cells by Plasma Exchange in Patients With Certain Autoimmune Neurological Diseases

**DOI:** 10.1002/iid3.70369

**Published:** 2026-03-07

**Authors:** Wanquan Xu, Shuting Chai, Gang Liu, Fei Tian, Weibi Chen, Dawei Shan, Yan Zhang

**Affiliations:** ^1^ Department of Neurology, Xuanwu Hospital Capital Medical University Beijing China; ^2^ Department of Neurology The First Hospital of Putian Putian City Fujian Province China

**Keywords:** autoimmune neurological diseases, cytokines, immunoglobulins, lymphocyte subsets, therapeutic plasma exchange

## Abstract

**Objective:**

Therapeutic plasma exchange (TPE) plays a crucial role in the management of autoimmune neurological disorders, although its effects on cytokines and immune cells are not fully understood. This study aimed to investigate the impact of TPE on immune cell subsets, cytokine levels, and immunoglobulins.

**Methods:**

A total of 85 patients with certain autoimmune neurological disorders underwent TPE, during which laboratory markers and complications were monitored. Blood and cerebrospinal fluid (CSF) samples were collected before and after TPE to assess changes in immunoglobulins, lymphocyte subsets, and cytokines. Clinical outcomes were evaluated 1 month after the TPE course using the modified Rankin Scale.

**Results:**

Among 408 TPE procedures, complications were mostly mild and manageable, with no fatalities. Peripheral blood leukocyte counts increased significantly, predominantly due to neutrophil elevation. Lymphocyte subset analysis revealed an increase in the proportion of T cells and a decrease in NK cells. Cytokine analysis showed a reduction in serum levels of IL‐6 and TNF‐α, and a decrease in CSF levels of IL‐6, IL‐8, IL‐10, and IFN‐γ. After TPE, immunoglobulin levels (IgA, IgG, and IgM) were significantly lower in both blood and CSF.

**Conclusion:**

TPE is a safe and well‐tolerated procedure for patients with certain autoimmune neurological disorders, potentially promoting recovery by modulating immune cell subsets and clearing pro‐inflammatory cytokines.

## 1 Introduction

Autoimmune neurological diseases are a group of autoimmune disorders that affect both the central nervous system (CNS) and the peripheral nervous system. These conditions are characterized by aberrant immune responses targeting antigens within the nervous system [1], resulting in a wide range of neurological symptoms. In severe cases, these disorders can pose a significant threat to life. In such clinical contexts, there is a critical need for prompt, effective, and safe therapeutic options, with therapeutic plasma exchange (TPE) emerging as a potential treatment. TPE is defined by the American Society for Apheresis (ASFA) 2023 guideline as “Plasma of the patient is separated from other components of blood, either by membrane filtration (mTPE) or centrifugation (cTPE). The plasma is removed with subsequent substitution of a replacement solution (e.g., human albumin and/or plasma) or a combination of crystalloid/colloid solution” [2]. According to the current ASFA guidelines, TPE is commonly employed in the treatment of certain neurological disorders such as Guillain‐Barré syndrome (GBS), neuromyelitis optica spectrum disorder (NMOSD), myasthenia gravis (MG), anti‐N‐methyl‐D‐aspartate (anti‐NMDA) receptor encephalitis, and chronic inflammatory demyelinating polyneuropathy (CIDP) [2].

While evidence suggests that TPE may exert therapeutic effects, including the removal of pathological substances from the plasma, such as pathogenic autoantibodies and immune complexes, as well as other pathogenic plasma components, it also affects lymphocyte proliferation and function, rendering these cells more responsive to immunosuppressants and chemotherapeutic agents. Additionally, TPE can modify the quantity and activation of B and T cells, enhance the function of regulatory T cells, and alter the ratio of T helper cells 1/2 (Th 1/Th 2) [3].

Cytokines are a class of small, soluble polypeptides secreted by immune and tissue cells that play crucial roles in development, homeostasis, and immune regulation. Based on their functions, cytokines are classified into several groups, including interleukins, interferons, tumor necrosis factors, colony‐stimulating factors, chemokines, and growth factors. Cytokines play a significant role in the pathogenesis of autoimmune neurological diseases [4], exerting a range of effects on various inflammatory cells. Most cytokines exhibit distinct characteristics and are elevated in many neuroimmune diseases [5, 6]. As a result, cytokines have emerged as valuable biomarkers for diagnosing autoimmune diseases, detecting intrathecal inflammation, and assessing both disease activity and therapeutic efficacy [6, 7]. However, the changes in cytokine levels in plasma and cerebrospinal fluid (CSF) during TPE for autoimmune neurological diseases remain inadequately understood. Furthermore, studies investigating cytokine alterations before and after TPE treatment in patients with autoimmune neurological diseases have predominantly focused on specific conditions [3].

Therefore, the aim of this study was to investigate the impact of TPE on various immunological parameters, including lymphocyte subsets, cytokines, and immunoglobulins.

## 2 Materials and Methods

### 2.1 Patient Eligibility

This study prospectively enrolled patients with certain autoimmune neurological diseases, particularly in the acute phase when there is no response to pharmacological immunotherapy or when there is insufficient time to await the effects of traditional medications, thereby necessitating urgent TPE. The patients were admitted to the Neurological department of Xuanwu Hospital, Capital Medical University, from January 2020 to June 2024. The inclusion criteria were as follows: (1) Age > 14 years; (2) In accordance with the 2019 ASFA guidelines [8], and the best practice recommendations [9], we selected patients with autoimmune neurological diseases for TPE by recognizing their distinct clinical symptoms, notable laboratory findings, atypical imaging results, and by ruling out other possible diagnose; (3) Patients provided voluntary informed consent. If the patients were unable to express their consent or sign the informed consent form, a close relative signed on their behalf. The exclusion criteria were as follows: (1) History of severe allergic reactions to plasma, human serum albumin, heparin, plasma separators, dialysis circuits, membranes, or tubing used in adsorption columns; (2) Irreversible circulatory failure; (3) Unstable myocardial infarction or ischemic stroke; (4) Intracranial hemorrhage or severe cerebral edema with brain herniation; (5) Presence of mental disorders preventing cooperation with the treatment.

### 2.2 Treatment Protocol

Our study involved patients with autoimmune encephalitis, NMOSD, acute disseminated encephalomyelitis, CIDP, and MG. These patients were either unresponsive to glucocorticoid immunotherapy (methylprednisolone 1 g/day for 3–5 days, with no clear effect after the last dose) or had contraindications to it, leading us to administer TPE. To minimize the impact of these treatments on the laboratory parameters of our study, we strategically administered intravenous immunoglobulin (IVIG), second‐line immunotherapy, and initiated long‐term immunotherapy. These interventions were carefully tailored to the specific disease and treatment context and were initiated after the completion of TPE and the collection of all necessary laboratory data. The sequence of treatments was based on the clinical guidelines and best practices in managing these autoimmune conditions [9, 10, 11, 12, 13]. For GBS patients, TPE is prioritized. Additionally, patients whose condition continues to progress or exhibits symptom fluctuations after TPE may receive IVIG treatment based on individual clinical assessment [14, 15].

TPE treatments were performed using the Jianfan Hemodiafiltration Machine and the PE‐08 plasma separator. The TPE circuit and plasma separator were primed with heparinized saline. The replacement fluids consisted of 5% albumin, physiological saline, and fresh frozen plasma. The volume exchanged was 1 plasma volume for each procedure. We calculated the estimated plasma volume (EPV) for each patient using the Kaplan formula, based on their hematocrit (Hct) level and body weight: EPV = [0.065 × weight (kg)] × [1 − Hct] [16]. Temporary vascular access was established via a double‐lumen, large‐caliber venous catheter in the femoral vein. The blood flow rate during TPE was maintained at 130–150 mL/min, with the plasma separation rate set at 10–25 mL/min. The plasma reinfusion temperature was kept at 36.5°C, and heparin anticoagulation was used throughout the procedure. Each TPE session was spaced 1–2 days apart, with 3–5 sessions constituting one treatment course.

### 2.3 Data Collected

We prospectively collected clinical data, including sex, age, and diagnosis, from patients with certain autoimmune neurological diseases who underwent TPE. Hematological counts, coagulation profiles, and serum levels of potassium, sodium, and albumin were recorded before and after each TPE session. Vital signs monitored during TPE included blood pressure, heart rate, peripheral oxygen saturation, respiratory rate, and body temperature. Technical parameters such as heparin dosage, plasma separation volume, blood flow rate, transmembrane pressure, arterial pressure, and venous pressure were closely monitored. Potential side effects during TPE, including allergic reactions, rashes, bleeding, and thrombosis, were also observed. Complications were classified according to the World Apheresis Association (WAA) criteria into mild (self‐limiting without treatment), moderate (requiring additional medications during TPE), severe (resulting in discontinuation of TPE), and death [17]. Blood lymphocyte subsets (CD3+ T cells %, CD4+ T cells %, CD8+ T cells %, CD4/CD8 ratio, CD19+ B cells %, CD20+ B cells %, CD16+56+ natural killer [NK] cells %) were assessed by flow cytometry before TPE and the day after the final TPE session.

We prospectively collected levels of cytokines (IL‐2, IL‐4, IL‐5, IL‐6, IL‐8, IL‐1β, IL‐17A, IL‐10, TNF‐α, IFN‐α, IL‐12p70, IFN‐γ) and immunoglobulins (IgA, IgG, IgM) in blood and CSF before and the day after the final TPE session. Cytokines were measured using flow cytometry, while immunoglobulins were measured using kinetic nephelometry.

### 2.4 The Efficacy of TPE After 1 Month

The efficacy of TPE was evaluated using the modified Rankin Scale (mRS) [18, 19], both prior to TPE and 1 month post‐treatment. A decrease of one point in the mRS score was considered an indication of clinical improvement.

### 2.5 Statistical Analysis

Statistical analyses were performed using SPSS version 27.0 (IBM Corp, Armonk, NY, USA). Continuous variables were expressed as means (± standard deviation [SD]) or medians (and interquartile ranges [IQRs]). Categorical data were presented as counts (percentages). Differences in categorical variables were assessed using the Pearson chi‐square test. Paired *t*‐tests were used for continuous variables, and Wilcoxon signed‐rank tests were applied for non‐parametric data to compare pre‐ and post‐TPE changes. All tests were two‐tailed, with a *p*‐value of < 0.05 considered statistically significant.

## 3 Results

### 3.1 Patient Characteristics

A total of 85 patients with certain autoimmune neurological diseases (Table 1) underwent 408 TPE procedures, consisting of 38 males (44.7%) and 47 females (55.3%), with a median age of 35 years and an IQR of 23–54 years. Anti‐NMDAR encephalitis was the most prevalent disease group among the patients, with 31 cases including 19 females, and a median age of 24 years. Patients with mRS scores ranging from 3 to 5 (moderate to severe disability) accounted for 94.12%.

**Table 1:** Demographic and clinical characteristics of patients with autoimmune neuroinflammatory diseases.

	Total (*n* = 85)
Male, *n* (%)	38 (44.7)
Female, *n* (%)	47 (55.3)
Age (years), median (IQR, range)	35 (31, 67)
Diagnosis, *n* (%)	
Autoimmune encephalitis	54 (63.53)
Anti‐NMDAR encephalitis	31 (36.74)
Autoimmune GFAP astrocytopathy	6 (7.06)
Anti‐GABAbR encephalitis	2 (2.35)
Anti‐GAD65 encephalitis	2 (2.35)
Anti‐LGI‐1 encephalitis	1 (1.18)
MOG antibody‐associated disease	1 (1.18)
Antibody‐negative autoimmune encephalitis	11 (12.94)
Neuromyelitis optica spectrum disorder	8 (9.41)
Myasthenia gravis	9 (10.59)
Guillain‐Barré syndrome	7 (8.24)
Chronic inflammatory demyelinating polyneuropathy	3 (3.53)
Acute disseminated encephalomyelitis	3 (3.53)
Neuropsychiatric systemic lupus erythematosus	1 (1.18)

Abbreviations: GABAbR, gamma‐aminobutyric acid receptor type b; GAD65, glutamate decarboxylase 65; GFAP, glial fibrillary acidic protein; IQR, interquartile range; LGI‐1, leucine‐rich glioma inactivated 1; MOG, myelin‐oligodendrocyte glycoprotein; NMDAR, N‐methyl‐D‐aspartate receptor.

### 3.2 Clinical Response to TPE

**Figure 1:**
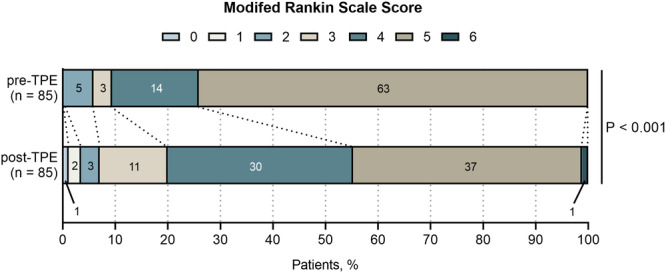
Comparison of mRS shift analysis: pre‐TPE versus 1 month post‐TPE. Compared with pre‐TPE, there was a statistically significant difference in the distribution of mRS scores 1 month after TPE. mRS, modified Rankin Scale; TPE, therapeutic plasma exchange.

Of the 85 patients, 34 (40%) demonstrated clinical improvement 1 month after TPE, as evidenced by a decrease of at least one point in the mRS score. There was a statistically significant difference in the mRS score distribution before and after TPE (*p* < 0.001) (Figure 1).

### 3.3 Adverse Events Associated With TPE

A total of 408 TPE procedures were performed across the 85 patients, among them, 20 patients (23.53%) were associated with no complications, while 65 (76.47%) patients experienced varying degrees of complications (Table 2). The most common complication was hypotension, observed in 72 procedures (17.65%), followed by mild hypokalemia and mild hyponatremia, which together occurred in 40 procedures (9.80%). In two cases, persistent thrombocytopenia during TPE led to immediate serum collection and platelet factor 4 antibody testing, which confirmed the diagnosis of heparin‐induced thrombocytopenia (HIT) type II. Following the discontinuation of heparin, platelet counts gradually increased, and no new thrombotic events or bleeding complications occurred. Severe complications, including plasma separator membrane rupture, occurred in two cases (2.35%), requiring termination of the procedure. No fatal TPE‐related adverse events were reported across the 408 procedures.

**Table 2:** Type, number, and percentage of complications with severity according to the World Apheresis Association grading scale in course of TPE.

Grading of adverse event	Symptoms/findings	Patients with complications, *n* = 85 (%)	TPE with complications, *n* = 408 (%)
Mild	Hypotension without drug intervention	11 (12.94)	13 (3.19)
	Increased involuntary movements or agitation	4 (4.71)	4 (0.98)
Modera	Allergic skin reaction	5 (5.88)	7 (1.72)
	Hypotension requiring drug intervention	19 (22.35)	72 (17.65)
	Tachycardia	4 (4.71)	4 (0.98)
	Bleeding at the venous cannula	1 (1.18)	1 (0.25)
	Increased involuntary movements or agitation	12 (14.12)	19 (4.66)
	Catheter‐related venous thrombosis	1 (1.18)	1 (0.25)
	Painful Spasm	1 (1.18)	3 (0.74)
	Hypokalemia (serum potassium < 3.5 mmol/L)	11 (12.94)	12 (2.94)
	Hyponatremia (serum sodium < 135 mmol/L)	18 (21.18)	28 (6.86)
	HIT Ⅱ	2 (2.35)	2 (0.49)
Severe	Plasma separator filter membrane	2 (2.35)	2 (0.49)

Abbreviations: HIT Ⅱ, heparin‐induced thrombocytopenia type Ⅱ; WAA, World Apheresis Association.

### 3.4 Leukocyte and Lymphocyte Subsets Before and After TPE

**Figure 2:**
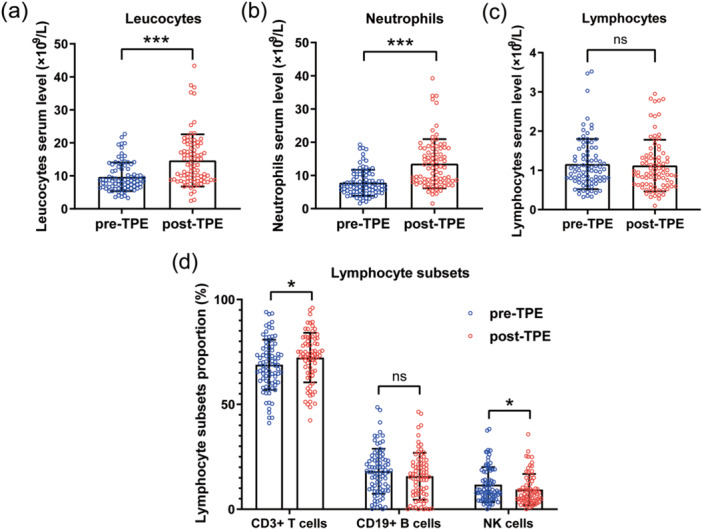
Changes in major immune cell populations associated with TPE. (a–c) Boxplots illustrating changes in peripheral blood leukocytes, neutrophils, and lymphocytes before and after TPE, with analysis conducted using the Wilcoxon signed‐rank test. (d) Changes in the proportion of CD3+ T lymphocytes, CD19+ B lymphocytes, and natural killer (NK) cells among lymphocyte subsets are shown, with results presented as mean ± SD, analyzed using the paired *t*‐test. *p < 0.05, ***p < 0.001; ns, not significant.

The changes in leukocyte and lymphocyte subsets following each TPE course, compared to the baseline values before TPE, are presented in Table 3. Figure 2 illustrates the changes in major immune cell populations associated with TPE. The median leukocyte count significantly increased after TPE (13.11 × 10^9^/L vs. 8.66 × 10^9^/L), with a statistically significant difference (*p* < 0.001). This increase was primarily attributed to a rise in neutrophil counts (*p* < 0.001), while lymphocyte counts did not exhibit a statistically significant difference. However, analysis of the changes in blood lymphocyte subsets revealed an increased proportion of T cells (*p* = 0.016) and a decreased proportion of NK cells (*p* = 0.044) after TPE.

**Table 3:** Changes in leukocytes and lymphocyte subsets before and after TPE.

	Pre‐TPE	Post‐TPE	*p* value
Leukocyte (×10^9^/L), median (IQR)	8.66 (6.56, 12.41)	13.39 (9.03, 19.69)	< 0.001^a^, ^c^
Neutrophil (×10^9^/L), median (IQR)	6.77 (5.03, 10.22)	12.26 (7.94, 18.15)	< 0.001^a^, ^c^
Lymphocyte (×10^9^/L), median (IQR)	1.00 (0.75, 1.52)	0.94 (0.64, 1.36)	0.531^a^
Each lymphocyte subset in the lymphocytes			
CD3+ T cells (%), mean ± SD	69.58 ± 11.78	72.35 ± 11.94	0.016^b^,^c^
CD4+ T cells (%), mean ± SD	38.42 ± 9.94	40.07 ± 10.21	0.174^b^
CD8+ T cells (%), mean ± SD	28.35 ± 10.55	29.64 ± 9.06	0.400^b^
CD4/CD8 ratio, mean ± SD	1.57 ± 0.78	1.47 ± 0.71	0.301^b^
CD19+ B cells (%), median (IQR)	17.73 (9.60, 23.95)	15.70 (7.00, 21.96)	0.214^a^
CD20+ B cells (%), median (IQR)	14.50 (5.10, 20.00)	13.20 (3.85, 19.85)	0.914^a^
CD16+56+ NK cells (%), median (IQR)	8.40 (5.40, 18.22)	7.20 (4.30, 13.40)	0.044^a^, ^c^

Abbreviations: CD4/CD8 ratios mean the ratio between the percentages of CD4 and CD8 cells; IQR, interquartile range; SD, standard deviation.

^a^ Paired sample *t*‐test.

^b^ Wilcoxon signed‐rank test.

^c^ Significance of differences.

### 3.5 Cytokine Profiles in Blood and CSF Before and After TPE

**Figure 3:**
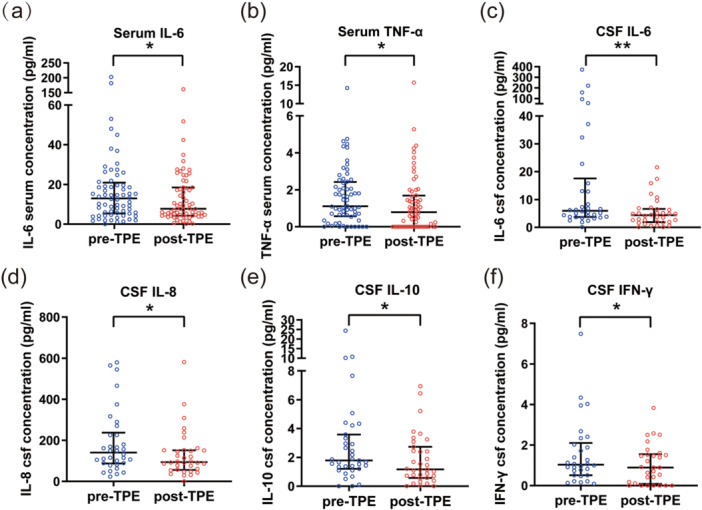
Changes in cytokine levels in plasma and CSF associated with TPE. (a) and (b) Serum levels of IL‐6 and TNF‐α in 51 patients, with blood cytokine measurements taken before (pre‐TPE, black bars) and after (post‐TPE, white bars) the TPE course. (c–f) CSF levels of IL‐6, IL‐8, IL‐10, and IFN‐γ in 35 patients, with cytokine measurements taken before (pre‐TPE, black bars) and after (post‐TPE, white bars) the TPE course. Results are presented as median and interquartile range. *p < 0.05, **p < 0.01.

In the actual clinical setting, cytokine levels were comprehensively evaluated in the blood of 51 patients and in the CSF of 35 patients, out of a total of 85 patients, both before and after TPE. The results were summarized in Table 4, and statistically significant changes were depicted in Figure 3. A significant decrease in serum IL‐6 and TNF‐α levels was observed post‐TPE (*p* < 0.05). Moreover, there was a marked reduction in CSF IL‐6 levels, with values dropping from 7.08 (3.99, 30.78) pg/mL to 4.85 (2.15, 8.94) pg/mL (*p* = 0.001). Decreased levels of IL‐8, IL‐10, and IFN‐γ were also noted in the CSF (*p* < 0.05).

**Table 4:** Changes in cytokine levels in serum and cerebrospinal fluid before and after TPE.

Cytokines	Serum (*n* = 51)			Cerebrospinal fluid (*n* = 35)		
	Pre‐TPE	Post‐TPE	*p* value	Pre‐TPE	Post‐TPE	*p* value
IL‐2 (pg/mL)	0.69 (0.22, 1.36)	0.78 (0, 1.36)	0.949	0.75 (0.02, 1.24)	0.41 (0, 1.59)	0.940
IL‐4 (pg/mL)	1.05 (0.17, 2.13)	0.89 (0, 2.33)	0.324	0.87 (0, 1.69)	0.48 (0, 1.61)	0.220
IL‐5 (pg/mL)	0.42 (0.01, 0.87)	0.32 (0, 0.93)	0.944	0.47 (0, 0.75)	0.34 (0, 0.68)	0.230
IL‐6 (pg/mL)	11.86 (5.03, 20.45)	7.77 (4.23, 20.11)	0.043^a^	7.08 (3.99, 30.78)	4.85 (2.15, 8.94)	0.001^a^
IL‐8 (pg/mL)	6.43 (3.37, 12.18)	7.86 (3.99, 10.33)	0.613	138.78 (89.24, 275.13)	113.59 (57.84, 200.76)	0.013^a^
IL‐1β (pg/mL)	0.83 (0.14, 1.60)	0.80 (0, 1.56)	0.371	0.41 (0, 1.49)	0.19 (0, 1.44)	0.681
IL‐17A (pg/mL)	5.00 (0, 9.39)	3.92 (0, 8.00)	0.166	4.64 (0, 9.26)	3.02 (0, 7.63)	0.108
IL‐10 (pg/mL)	3.47 (1.71, 5.25)	4.01 (1.86, 8.43)	0.790	1.78 (1.13, 3.45)	1.34 (0.59, 3.21)	0.017^a^
TNF‐α (pg/mL)	1.00 (0.22, 2.27)	0.73 (0, 1.69)	0.031^a^	0.98 (0, 1.94)	0.77 (0, 2.02)	0.086
IFN‐α (pg/mL)	0.72 (0.10, 1.47)	0.58 (0, 1.24)	0.418	0.33 (0, 1.07)	0.09 (0, 1.40)	0.758
IL‐12p70 (pg/mL)	1.03 (0, 2.32)	0.61 (0, 2.47)	0.371	1.01 (0, 1.81)	0.55 (0, 2.22)	0.434
IFN‐γ (pg/mL)	1.19 (0.40, 2.21)	0.75 (0.01, 1.82)	0.161	0.91 (0.15, 2.18)	0.67 (0, 1.63)	0.021^a^

*Note:* Values are given as median (interquartile range), and the Wilcoxon signed‐rank test was used for analysis.

Abbreviations: IFN, interferon; IL, interleukin; TNF, tumor necrosis factor.

^a^ Significance of differences.

### 3.6 Immunoglobulins in Blood and CSF Before and After TPE

**Figure 4:**
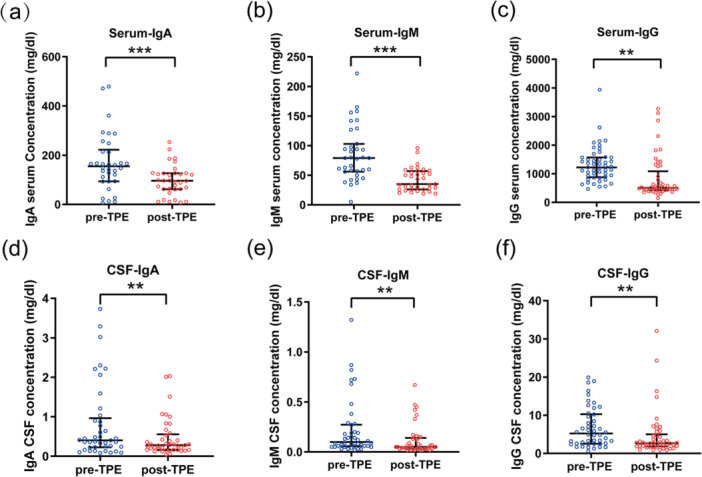
Absolute changes in immunoglobulin levels in serum and cerebrospinal fluid associated with TPE. (a–c) Evaluation of serum IgA, IgM, and IgG levels before (pre‐TPE) and after (post‐TPE) TPE. (d–f) Evaluation of cerebrospinal fluid (CSF) IgA, IgM, and IgG levels before (pre‐TPE) and after (post‐TPE) TPE. Results are expressed as median and interquartile range. The Wilcoxon signed‐rank test was used for statistical analysis. Post‐TPE, immediately after therapeutic plasma exchange; pre‐TPE, prior to therapeutic plasma exchange. **p < 0.01, ***p < 0.001.

As shown in Figure 4, there was a significant decrease in the levels of IgA, IgM, and IgG in both the blood and CSF following TPE.

## 4 Discussion

In this prospective study involving 85 patients with certain autoimmune neurological diseases, among whom 94.12% had severe conditions with mRS scores of 3–5, we observed that 40% of patients demonstrated clinical symptom improvement within a short‐term period of 1 month following TPE. Although evidence from randomized clinical trials is limited, published case series experiences suggest that TPE plays a significant role in improving the clinical conditions of these patients. Previous studies have shown that TPE can reduce the duration and severity of symptoms associated with GBS, and in some cases, it has been shown to prevent the need for mechanical ventilation [20]. Additionally, TPE is indicated for patients with moderate to severe MG who do not respond to immunosuppressants and anticholinesterase inhibitors, as well as for those in myasthenic crisis [21, 22]. Furthermore, TPE is also used in the treatment of anti‐NMDA receptor encephalitis, along with other autoimmune encephalitides caused by autoantibodies targeting neuronal surfaces [23, 24]. In our center's previous clinical observational studies, TPE was shown to rapidly improve clinical manifestations in patients with severe anti‐NMDA receptor encephalitis who did not respond to steroids and IVIGs [25]. These findings highlight that TPE is an effective and, at times, essential treatment modality for certain autoimmune neurological diseases, particularly in the acute phase when there is no response to pharmacological immunotherapy or when there is insufficient time to await the effects of traditional medications, thereby necessitating urgent TPE. Concurrently, as an invasive treatment, TPE in our study was associated with minor and controllable complications, with no life‐threatening complications or deaths recorded throughout the procedure.

In our study, we conducted a comparative analysis of leukocyte counts before and after TPE, revealing a significant increase, primarily in neutrophil counts. This finding aligns with a previous study, which observed leukocytosis in 42.7% of patients following TPE [26]. Clinically, these patients often lack overt signs of infection such as fever, clinical symptoms, or elevated laboratory markers. Typically, dynamic monitoring during subsequent follow‐ups showed a gradual decrease in leukocyte counts to normal levels a few days later. The underlying mechanisms for this phenomenon are thought to involve a stress response induced by the invasive nature of TPE. These factors may lead to temporary reactive leukocytosis and the release of cells from the spleen, bone marrow, and vascular walls into the peripheral blood, thus increasing peripheral leukocyte counts.

In patients with certain autoimmune neurological disorders, we monitored changes in lymphocyte subsets following TPE. We observed an increase in the proportion of T lymphocytes and a decrease in NK cell subsets post‐TPE. Alterations in lymphocyte subsets are critical indicators of immune dysregulation in autoimmune neurological disorders. Previous studies have demonstrated the impact of TPE on lymphocyte subsets, leading some researchers to propose that these effects constitute a significant mechanism of action for TPE [27, 28]. Two studies assessing lymphocyte quantification and functionality in patients with GBS undergoing TPE revealed that, compared to healthy controls, GBS patients had reduced T cell counts and increased B cell counts prior to TPE. Post‐TPE, T cell counts increased while B cell counts decreased, thereby restoring the distribution of these cells closer to that observed in healthy controls [28, 29]. It has been hypothesized that the improvement in clinical symptoms following TPE may be associated with the normalization of T and B lymphocyte subsets.

Additionally, a reduction in NK cell proportion was observed post‐TPE. NK cells are composed of various subsets with different cytotoxic activities, and they play a pivotal role in the initiation, progression, and resolution of autoimmune diseases [30, 31]. In a study evaluating the effect of TPE on the cytotoxicity of peripheral blood NK cells in patients with MG, a decrease in NK cell cytotoxicity was noted following TPE, although no significant change in the percentage of NK cells was observed. The researchers concluded that the decrease in cytotoxic activity was not due to a reduction in the number of NK cells [32]. Our study showed a decrease in the proportion of NK cells after TPE; however, further investigation is required to examine potential changes in NK cell cytotoxic activity.

In our study, we also observed a reduction in peripheral blood levels of the pro‐inflammatory cytokines IL‐6 and TNF‐α following TPE. However, similar results were not consistently reported in previous studies. A comparative study of immunoadsorption and TPE in patients with neurological autoimmune diseases found an increase in pro‐inflammatory cytokines (IL‐12, IL‐17, IL‐6, and TNF‐α) post‐TPE, while a significant decrease was observed with Immunoadsorption [33]. One possible explanation for the lack of reduction after TPE in this study is the potential activation of cytokine production by exogenous fresh frozen plasma or albumin [33]. Additionally, a study measuring IL‐6, IL‐1β, and TNF‐α in severe sepsis patients before and after TPE showed no effect of TPE on cytokine levels in plasma [34]. Nevertheless, the removal of cytokines has been suggested as a potential mechanism of action for TPE [3, 27]. Our study's findings, showing a reduction in cytokine levels post‐TPE, support this hypothesis, and such changes may play a positive role in improving clinical symptoms.

Neuroinflammation is a complex process that plays a central role in the pathogenesis of autoimmune neurological diseases. Cytokines are key modulators of neuroinflammation and significantly influence disease mechanisms [4]. Cytokines and chemokines exert diverse effects on various inflammatory cells, many of which are elevated in numerous neuroimmune diseases. IL‐6 is crucial for the differentiation of T cells into Th17 cells, contributes to tissue regeneration, inflammation, and pathogen resistance, and plays a central role in neuroinflammatory pathways within the CNS [4, 35]. The integrity of the blood–brain barrier (BBB) is critical for the homeostasis of the CNS, regulating the bidirectional exchange of fluids and solutes between the peripheral blood and the CNS microenvironment. Previous research has demonstrated a strong correlation between BBB disruption and elevated levels of IL‐6 and TNF‐α. The compromised integrity of the BBB is implicated in the development and progression of numerous neuroinflammatory conditions [36, 37, 38]. Within the context of NMOSD, in vitro and ex vivo BBB models demonstrated that blocking IL‐6 suppressed the NMO‐IgG‐induced transmigration of T cells and barrier dysfunction. In the in vivo study, blocking IL‐6 signaling suppressed the migration of T cells into the spinal cord and prevented the increased BBB permeability [39]. TNF‐α is involved in neuroplasticity and myelination, although at pathological levels, it might cause excitotoxicity, neuroinflammation, and breakdown of the BBB [40]. It is a critical factor in the pathogenesis of various disorders, and pharmacological targeting of TNF‐α has shown clinical benefits [41, 42]. Thus, the reduction in IL‐6 and TNF‐α levels in peripheral blood may promote the repair of the BBB and aid in the recovery process of the autoimmune neurological diseases.

There have been few studies conducted on the impact of TPE on cytokine levels in the CNS. In this study, we measured CSF cytokine levels before and after TPE, observing a decrease in the pro‐inflammatory cytokines IL‐6, IL‐8, and IFN‐γ, alongside a reduction in the anti‐inflammatory cytokine IL‐10. Pro‐inflammatory cytokines play a crucial role in activating microglia and astrocytes during the pathogenesis of autoimmune neurological diseases, leading to the release of additional pro‐inflammatory mediators and chemokines, which may exacerbate neuroinflammation and promote neurodegeneration. Conversely, anti‐inflammatory cytokines help suppress neuroinflammation and promote neuronal survival [4]. IL‐6 is a key cytokine in CNS inflammatory diseases, with a wide range of functions and biological activities. It contributes to acute inflammation by inducing the synthesis of acute‐phase proteins, and thus, an elevated IL‐6 concentration in the CSF can serve as a non‐specific marker of CNS inflammation [6, 43]. Additionally, IL‐6 acts as a B cell stimulatory factor, promoting the differentiation of B cells into plasma cells and subsequent immunoglobulin production [44]. IL‐8 is an inflammatory chemokine that attracts and activates neutrophils and lymphocyte subsets, while also exhibiting potent angiogenic effects. Produced by various cell types, including monocytes, lymphocytes, granulocytes, fibroblasts, endothelial cells, and astrocytes, IL‐8 levels in the CSF increase during CNS inflammation [45, 46]. IFN‐γ is a pro‐inflammatory cytokine primarily produced by peripheral cells, such as T lymphocytes, NK cells, and NK T cells, and it can also be synthesized by cells within the CNS under specific stimuli [47]. IL‐10, recognized as a quintessential anti‐inflammatory cytokine produced by glial cells in the CNS, exhibits a delayed response that is attributed to the presence of inflammatory mediators [48, 49]. Additionally, the inflammatory cytokines IL‐6 and TNF‐α have been demonstrated to induce IL‐10 production by microglia in a dose‐dependent manner [50]. TPE may reduce the signals that prompt glial cells in the CNS to produce IL‐10, thereby leading to a decrease in IL‐10 levels. A meta‐analysis demonstrated higher concentrations of IL‐6, IL‐8, and IL‐10 in the CSF of patients with encephalitis compared to controls, and a trend toward increased IFN‐γ concentrations was also observed in the CSF of 252 patients with encephalitis [51]. The observed decrease in these cytokine levels suggests that TPE may effectively clear inflammatory mediators, thereby contributing to immune system regulation and restoring a relatively balanced state. These changes are generally beneficial for the clinical management of autoimmune neurological diseases, suggesting that TPE may aid in alleviating disease progression and symptoms.

TPE works by directly removing immunoglobulins from the bloodstream through a physical process, particularly autoantibodies associated with the disease. This mechanism forms the basis of TPE, but it also results in a reduction of overall immunoglobulin levels, a finding observed in both this study and previous research [52]. However, there is a lack of studies addressing changes in CSF immunoglobulin levels. Therefore, this study also assessed CSF immunoglobulin levels before and after TPE. Similar to the peripheral blood circulation, we observed a decrease in immunoglobulin levels within the CSF. In patients with autoimmune CNS diseases, the permeability of the BBB is often altered, which facilitates the movement of immunoglobulins from the CSF into the bloodstream. During TPE, some of these immunoglobulins may be removed along with the blood circulation. Additionally, TPE may modulate systemic immune responses, such as reducing inflammatory mediators and cytokines, which could further influence immunoglobulin levels in the CSF.

This study has several limitations. First, the sample was derived from a single center with a relatively small sample size, and only pre‐ and post‐TPE changes in indicators were assessed, with no measurement at 1‐month follow‐up to reflect the immunoglobulin related rebound effect after TPE on short‐term TPE efficacy, without a control group to account for the potential effects of the disease itself or other treatments on the observed changes. Second, due to the limited patient sample size and the actual clinical practice of collected levels of cytokines and immunoglobulins, this study could not perform a detailed analysis of certain autoimmune neurological diseases and may have potential biases. Third, given the substitution with foreign immunoglobulin by adding fresh frozen plasma to the exchange volume during TPE, as well as the concomitant immunotherapy, the results regarding cytokine and immunoglobulin levels should be interpreted with caution. Further evaluation of cytokine and lymphocyte subset changes is needed in larger, more homogeneous disease phenotypes.

In conclusion, our study demonstrates that TPE is both effective and well‐tolerated in patients with certain autoimmune neurological disorders. The observed normalization of peripheral blood T and B lymphocyte subsets may be associated with the improvement of clinical symptoms. Additionally, the reduction in peripheral blood levels of IL‐6 and TNF‐α may contribute to the recovery of the BBB and the overall disease recuperation. Similarly, the decrease in CSF levels of IL‐6, IL‐8, IFN‐γ, and IL‐10 may reflect the reduction of neuroinflammation. These findings provide further scientific support for the therapeutic value of TPE in the management of autoimmune neurological diseases.

## Author Contributions

**Wanquan Xu:** conceptualization, writing – original draft, data curation, methodology, formal analysis, visualization, validation. **Shuting Chai:** conceptualization, writing – original draft, data curation, methodology, formal analysis, visualization, validation. **Gang Liu:** data curation. **Fei Tian:** data curation. **Weibi Chen:** data curation. **Dawei Shan:** data curation. **Yan Zhang:** writing – review and editing, supervision, validation. All authors have read and agreed to the published version of the manuscript.

## Ethics Statement

This study was approved by the Ethics Committee of Xuanwu Hospital, Capital Medical University, and adhered to the principles of the Declaration of Helsinki.

## Conflicts of Interest

The authors declare no conflicts of interest.

## Data Availability

Anonymized data from this study are available on reasonable request.
